# Comparison of the cardiometabolic profiles of adolescents conceived through ART with those of a non-ART cohort

**DOI:** 10.1093/humrep/deac122

**Published:** 2022-05-28

**Authors:** L A Wijs, D A Doherty, J A Keelan, P Burton, J L Yovich, L Beilin, T A Mori, R C Huang, L A Adams, J K Olynyk, O T Ayonrinde, B Penova-Veselinovic, R J Hart

**Affiliations:** Division of Obstetrics and Gynaecology, University of Western Australia, Perth, WA, Australia; Division of Obstetrics and Gynaecology, University of Western Australia, Perth, WA, Australia; Women and Infants Research Foundation, Perth, WA, Australia; Division of Obstetrics and Gynaecology, University of Western Australia, Perth, WA, Australia; Women and Infants Research Foundation, Perth, WA, Australia; School of Biomedical Sciences, University of Western Australia, Perth, WA, Australia; School of Medical and Health Sciences, Edith Cowan University, Perth, WA, Australia; Concept Fertility Centre, Perth, WA, Australia; School of Pharmacy and Biomedical Sciences, Curtin University, Perth, WA, Australia; PIVET Medical Centre, Perth, WA, Australia; Division of Internal Medicine, Medical School, University of Western Australia, Perth, WA, Australia; Division of Internal Medicine, Medical School, University of Western Australia, Perth, WA, Australia; Centre for Child Health Research, University of Western Australia, Perth, WA, Australia; Telethon Kids Institute, University of Western Australia, Perth, WA, Australia; Division of Internal Medicine, Medical School, University of Western Australia, Perth, WA, Australia; School of Medical and Health Sciences, Edith Cowan University, Perth, WA, Australia; Department of Gastroenterology & Hepatology, Fiona Stanley Hospital, Perth, WA, Australia; Division of Internal Medicine, Medical School, University of Western Australia, Perth, WA, Australia; Department of Gastroenterology & Hepatology, Fiona Stanley Hospital, Perth, WA, Australia; Faculty of Health Sciences, Curtin University, Perth, WA, Australia; Division of Obstetrics and Gynaecology, University of Western Australia, Perth, WA, Australia; Fertility Specialists of Western Australia, Perth, WA, Australia

**Keywords:** ART, IVF/ICSI outcome, long-term outcomes, cardiometabolic, cardiovascular

## Abstract

**STUDY QUESTION:**

Is the cardiometabolic health of adolescents conceived through ART worse than that of their counterparts conceived without ART?

**SUMMARY ANSWER:**

The majority of cardiometabolic and vascular health parameters of adolescents conceived through ART are similar or more favourable, than those of their counterparts of similar age and conceived without ART.

**WHAT IS KNOWN ALREADY:**

It has been proposed that the cardiometabolic health of offspring conceived with ART may be unfavourable compared to that of their counterparts conceived without ART. The literature pertaining to cardiometabolic health of offspring conceived after ART is contradictory, but generally suggests unfavourable cardiometabolic health parameters, such as an increase in blood pressure (BP), vascular dysfunction and adiposity, as well as unfavourable glucose and lipid profiles. With over 8 million children and adults born through ART worldwide, it is important to investigate whether these early signs of adverse cardiometabolic differences persist into adolescence and beyond.

**STUDY DESIGN, SIZE, DURATION:**

The Growing Up Healthy Study (GUHS) is a prospective cohort study that recruited 303 adolescents and young adults conceived after ART (aged 13–21 years) and born between 1991 and 2001 in Western Australia. Their health parameters, including cardiometabolic factors, were assessed and compared with counterparts from the Raine Study Generation 2 (Gen2). The 2868 Gen2 participants were born 1989–1992 and are representative of the Western Australian adolescent population. At ∼17 years of age (2013–2017), 163 GUHS participants replicated assessments previously completed by Gen2 at a similar age.

**PARTICIPANTS/MATERIALS, SETTING, METHODS:**

Cardiometabolic parameters were compared between a total of 163 GUHS and 1457 Gen2 adolescents. Separate male (GUHS n = 81, Gen2 n = 735) and female (GUHS n = 82, Gen2 n = 722) analyses were conducted. Assessments consisted of a detailed questionnaire including health, lifestyle and demographic parameters, anthropometric assessments (height, weight, BMI, waist circumference and skinfold thickness), fasting serum biochemistry, arterial stiffness and BP (assessed using applanation tonometry). Abdominal ultrasonography was used to assess the presence and severity of hepatic steatosis, and thickness of abdominal fat compartments. Non-alcoholic fatty liver disease (NAFLD) was diagnosed if there was sonographic fatty liver in the absence of significant alcohol consumption. Chi^2^, Fisher’s exact and Mann–Whitney *U* tests, performed in SPSS V25, examined cohort differences and generalized estimating equations adjusted for the following covariates: singleton vs non-singleton pregnancy, birthweight (z-score), gestational age, BMI, smoking, alcohol consumption in the past 6 months and parent cardiovascular status. Arterial stiffness measures and waist circumference were additionally adjusted for height, and female analyses were additionally adjusted for use of oral contraceptives in the preceding 6 months.

**MAIN RESULTS AND THE ROLE OF CHANCE:**

In adjusted analyses, GUHS females had a lower BMI (22.1 vs 23.3 kg/m^2^, *P* = 0.014), and thinner skinfolds (triceps, subscapular, mid-abdominal; 16.9 vs 18.7 mm, *P* = 0.021, 13.4 vs 15.0 mm, *P* = 0.027, 19.7 vs 23.2 mm, *P* < 0.001, respectively), whereas males were not significantly different. Waist circumference was lower in GUHS adolescents (males: 78.1 vs 81.3 cm, *P* = 0.008, females: 76.7 vs 83.3 cm, *P* = 0.007). There were no significant differences between the two groups in glucose, insulin, homeostatic model assessment for insulin resistance, low-density lipoprotein cholesterol, non-high-density lipoprotein cholesterol (non-HDL-C), total cholesterol (TC), alanine aminotransferase and high-sensitivity C-reactive protein in both sexes. In females, serum triglycerides were lower in GUHS adolescents (1.0 vs 1.2 mmol/l, *P* = 0.029). GUHS males had higher serum HDL-C (1.1 vs 1.0 mmol/l, *P* = 0.004) and a lower TC/HDL-C ratio (3.2 vs 3.6, *P* = 0.036). There were no significant differences in the prevalence of NAFLD or steatosis severity scores between the cohorts in males and females. GUHS females had less subcutaneous adipose tissue (9.4 vs 17.9 mm, *P* < 0.001), whereas GUHS males had greater visceral adipose thickness (44.7 vs 36.3 mm, *P* < 0.001). There was no significant difference in pre-peritoneal adipose thickness. Pulse wave velocity was lower in GUHS males (5.8 vs 6.3 m/s, *P* < 0.001) and heart rate corrected augmentation index was lower in GUHS females (−8.4 vs −2.7%, *P* = 0.048). There were no significant differences in BP or heart rate in males or females between the two groups.

**LIMITATIONS, REASONS FOR CAUTION:**

Despite the substantial study size and the unique study design of the ART cohort, we were unable to differentiate between different types of ART, due to the low number of ICSI cycles (e.g. IVF vs ICSI), draw definite conclusions, or relate the outcomes to the cause of infertility. Considering the differences in time points when both cohorts were studied, external factors could have changed, which could not be accounted for. Given the observational nature of this study, causation cannot be proven.

**WIDER IMPLICATIONS OF THE FINDINGS:**

Contrary to our hypothesis and previous findings focussing mainly on childhood, this study reports mostly similar or favourable cardiometabolic markers in adolescents conceived with ART compared to those conceived without ART. The greater visceral adipose thickness, particularly present in males, requires further investigation. While these findings are generally reassuring, future well-designed and appropriately powered studies are required to definitively address the issue of cardiometabolic health in ART adults.

**STUDY FUNDING/COMPETING INTEREST(S):**

This project was supported by NHMRC project grant number 1042269 and R.J.H. received education grant funding support from Ferring Pharmaceuticals. R.J.H. is the Medical Director of Fertility Specialists of Western Australia and a shareholder in Western IVF. He has received educational sponsorship from MSD, Merck-Serono and Ferring Pharmaceuticals. P.B. is the Scientific Director of Concept Fertility Centre, Subiaco, Western Australia. J.L.Y. is the Medical Director of PIVET Medical Centre, Perth, Western Australia.

**TRIAL REGISTRATION NUMBER:**

N/A.

## Introduction

Offspring conceived after ART may be at an increased risk of unfavourable cardiometabolic markers and health. The literature to date on cardiometabolic health of ART offspring is contradictory, but various studies have associated ART with unfavourable cardiometabolic health parameters, particularly in children. Studies in and beyond adolescence are scarce, and many studies focus only on one or a few cardiometabolic parameters per study. Childhood studies have reported an increase in systolic as well as diastolic blood pressure (BP; (Ceelen *et al.*, 2008; Sakka *et al.*, 2010; Hart and Norman 2013; Guo *et al.*, 2017), vascular dysfunction (Scherrer *et al.*, 2012; Meister *et al.*, 2018), adiposity (Ceelen *et al.*, 2007; Belva *et al.*, 2012; Hart and Norman, 2013), fasting glucose and insulin concentrations and an unfavourable lipid profile (Ceelen *et al.*, 2008; Sakka *et al.*, 2010; Hart and Norman 2013; Cui *et al.*, 2020) in those conceived with ART. As concluded by a grand theme review in 2019, ART offspring appears to be at an increased risk of hypertension, an unfavourable metabolic profile and cardiovascular dysfunction (Berntsen *et al.*, 2019). Certain cardiometabolic measures in adolescence are good predictors of cardiovascular disease later in life. Studies have suggested that pulse wave velocity (PWV), a measure of arterial stiffness, is an early predictor of atherosclerosis and cardiovascular disease (Zhong *et al.*, 2018). Furthermore, non-alcoholic fatty liver disease (NAFLD) can be viewed as the hepatic marker of metabolic syndrome (Marchesini *et al.*, 2001). An increase in markers of the metabolic syndrome in childhood and adolescence, such as central obesity, hypertension, glucose intolerance, insulin resistance and dyslipidaemia, often persist into adulthood and translate to an increased risk of diabetes and cardiovascular disease in adulthood (Bao *et al.*, 1994; Chen *et al.*, 2000). Despite some studies reporting an increased risk of unfavourable cardiometabolic and cardiovascular outcomes in offspring conceived after ART, other, recent studies do not demonstrate such an increased risk in those conceived after ART (Shiloh *et al.*, 2019; Juonala *et al.*, 2020; Mizrak *et al.*, 2022).

With one in 20 children in Australia currently born after conception through ART (Newman *et al.*, 2020), it is important to investigate whether the childhood differences in cardiometabolic parameters reported in some studies persist into adolescence and adulthood. To this end, we have established a cohort of adolescents conceived after ART in Western Australia: The Growing Up Healthy Study (GUHS). This cohort was established to compare their long-term health parameters to those of a representative control population conceived without ART from the Raine Study (Straker *et al.*, 2017). The objective of the present study was to compare cardiometabolic risk factors of adolescents conceived by ART with that of adolescents of similar age and sex conceived without ART.

## Materials and methods

### Study populations

#### ART cohort

**Figure 1.**
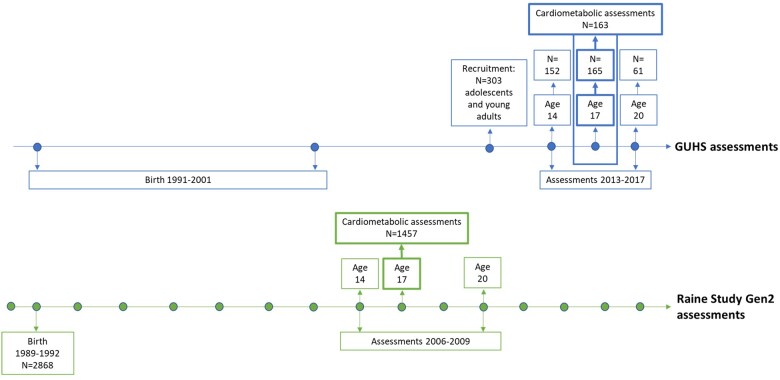
**Timeline of the GUHS and the Raine Study Gen2 recruitment and assessments, with cardiometabolic assessments at age 17.** GUHS, Growing Up Healthy Study; Gen2, Generation 2.

The GUHS cohort aimed to recruit all adolescents conceived through ART, born 1991–2001 in the two fertility clinics operating at the time: PIVET Medical Centre and Concept Fertility Centre in Perth, Western Australia, Australia. From 404 families recruited through the fertility clinics, 303 adolescents participated in the study. These adolescents completed various health assessments between 2013 and 2017. Depending on age at enrolment, participants completed assessments at one or two of the follow-ups conducted at ages 14, 17 and 20. The 17-year follow-up, including cardiometabolic assessments, was completed by 179 age-eligible GUHS participants. After the exclusion of 15 participants conceived through gamete intrafallopian transfer (GIFT), and one participant conceived after IUI, 163 GUHS participants (81 males and 82 females) were included in this study. These participants were conceived through IVF (n = 117) and ICSI (n = 35), of which n = 95 were fresh embryo transfers (ET) and n = 57 were frozen embryo transfers (FET). Type of ART status could not be confirmed in 11 participants. GUHS participants replicated assessments previously completed by adolescents of similar age and sex, conceived without ART, from the Raine Study, by following the same protocols and using the same equipment. The recruitment and assessment process is shown in Fig. 1.

#### Non-ART cohort

The Raine Study recruited pregnant women in Western Australia between 1989 and 1991 to investigate the safety and effects of ultrasound on the foetus (https://www.rainestudy.org.au; Newnham *et al.*, 1991). A total of 2900 women were enrolled around the 18th week of gestation at antenatal booking clinics. A final number of 2868 children born to 2804 mothers formed Generation 2 (Gen2) of the Raine Study. The Gen2 participants have completed assessments at regular intervals from birth to early adulthood, to investigate the effect of perinatal health on subsequent childhood and adult health (Straker *et al.*, 2017). The cohort has a current follow-up rate of 70%, including 1800 participants just over the age of 30. The Raine Study adolescents have been shown to be representative of the Western Australian adolescent population at the time of assessments (Robinson *et al.*, 2010). Cardiometabolic assessments of the 17-year follow-up were completed by 1467 Gen2 participants (2006–2009). After the exclusion of participants, five participants conceived after IVF and five participants conceived after GIFT, 1457 Gen2 participants (735 males and 722 females) were included in this study.

### Cardiovascular assessments

Comprehensive cardiometabolic profiles, including anthropometry, serum fasting biochemistry, a liver ultrasound, arterial stiffness and BP were compared between the two cohorts and analysed separately in males and females.

### Data sources and measurements

#### Anthropometric assessments

Standing height was measured using a stadiometer (to the nearest 0.1 cm; Wall mounted Holtain stadiometer, UK) and weight was measured seated, with participants lightly clothed, using an electronic scale (to the nearest 0.1 kg; Wedderburn Chair Scales, Australia). BMI was calculated (kg/m^2^) and obesity was defined following age- and gender-adjusted BMI criteria (Cole *et al.*, 2007). Waist circumference was measured twice at the umbilicus level, using a tape measure, and the average of both measurements was recorded (to the nearest 0.1 cm; Birch plastic measuring tape, Australia). Skinfold thickness was measured twice using a calliper, and the average of both measurements was recorded (triceps, subscapular, mid-abdominal and suprailiac skinfolds, to the nearest 0.1 mm; Holtain skinfold Callipers, UK).

#### Fasting serum biochemistry measures

Venous blood samples were collected after overnight fasting. Serum and plasma samples were stored at −80 degrees and analysed in the PathWest Laboratory, Perth, Western Australia, Australia. Serum biochemistry included glucose, insulin, triglycerides, low-density lipoprotein cholesterol (LDL-C), high-density lipoprotein cholesterol (HDL-C), total cholesterol (TC), TC/HDL-C ratio, non-HDL cholesterol (non-HDL-C), high-sensitivity C-reactive protein (hs-CRP; excluding CRP >10 mg/l indicating acute infection) and alanine aminotransaminase (ALT).

Homeostatic model assessment for insulin resistance (HOMA-IR) was calculated as: Fasting insulin (mU/l) × (fasting glucose (mmol/l)/22.5) (Matthews *et al.*, 1985).

#### Abdominal ultrasonography

Abdominal ultrasonography was conducted for investigation of the presence and severity of hepatic steatosis and measurement of the thickness of abdominal adipose tissue (mm) compartments (subcutaneous, visceral and pre-peritoneal), using a Siemens Antares ultrasound machine with a CH 6-2 curved array probe (Sequoia, Siemens Medical Solutions, USA). GUHS participants underwent abdominal ultrasonography according to the same protocol as participants from the Raine Study Gen2, previously described (Ayonrinde *et al.*, 2011).

Qualified sonographers performed focused liver ultrasonography in accordance with the protocol described by Hamaguchi *et al.* (2007), which provides 92% sensitivity and 100% specificity for the histological diagnosis of hepatic steatosis.

For both cohorts, a separate single specialist radiologist, who was blinded to the clinical and laboratory characteristics of the subjects, interpreted the ultrasound images. Scores of 0–3, 0–2 and 0–1 were determined from captured images for liver echotexture (bright liver and hepatorenal echo contrast), deep attenuation (diaphragm visibility) and vessel blurring (intrahepatic vessel visibility). The diagnosis of hepatic steatosis required a total score of ≥2, inclusive of echotexture score of ≥1. The severity of hepatic steatosis was classified by the total fatty liver score as 0–1 (no fatty liver), 2–3 (mild fatty liver) or 4–6 (moderate to severe fatty liver). Adolescents with sonographic fatty liver, the absence of self-reported long-term use of steatogenic medications and a self-reported weekly alcohol intake of <140 g for males and <70 g for females over the preceding year were classified as having NAFLD.

Abdominal (subcutaneous, visceral and pre-peritoneal) adipose thickness measurements were performed with previously described criteria that correlate closely with compartmental adipose areas and cardiovascular and metabolic risk factors (Liu *et al.*, 2003; Kim *et al.*, 2004; Denzer *et al.*, 2009).

#### Measures of BP and arterial stiffness

Systolic and diastolic BP were measured by oscillometric sphygmomanometer (DINAMAP vital signs monitor 8100, DINAMAP XL vital signs monitor or DINAMAP ProCare 100), after resting for 5 min and using the appropriate cuff size. Six readings were taken in a supine position, every 2 min for 10 min. The average value was calculated after excluding the first reading.

PWV (m/s) and heart rate corrected augmentation index (AI, %), as measures of arterial stiffness were measured using a SphygmoCor instrument (AtCor Medical Pulse Wave Analysis System SCOR-Px, Australia; Wilkinson *et al.*, 1998). GUHS participants replicated SphygmoCor assessments according to the same protocol as the Raine Study Gen2 participants, previously described (Huang *et al.*, 2013). After 5-min rest, three electrocardiogram leads were attached to the left leg, right arm and left arm. Tonometers were applied to two sites (the carotid artery and the distal dorsalis pedis). Distance measurement was recorded in millimetres between the manubrium sternum and the two sites. The pulse wave analysis was recorded from the supported radial artery with the wrist facing up. Data were entered after the waveform was maintained for 10 s and the test was repeated until at least two captures were recorded with a quality index of >80. PWV was calculated by dividing the distance between tonometers by the transit time of the arterial pulse wave. Heart rate corrected AI was defined as the difference in the second and first systolic pressure peaks as a percentage of pulse pressure at heart rate 75 beats per minute.

#### Questionnaires

Detailed questionnaires included tobacco smoking (smoked >10 cigarettes in their life and smoked in the past 4 weeks), alcohol intake (consumption in the past 6 months), exercise (hours per week), parent cardiovascular status (i.e. parent heart disease, diabetes, high cholesterol (unspecified) and arterial hypertension), use of medications (particularly oral contraceptive use in the past 6 months for females) and socio-economic status (SES), at date of assessment. SES was based on postcodes and is reported as advantage–disadvantage deciles based on the Socio-Economic Indexes for Areas (SEIFA) scores for Western Australia, from the Australian Bureau of Statistics (from lowest score of 1 to the highest score of 10; Australian Bureau of Statistics, 2006, 2011, 2016). For presentation, we transformed deciles into quintiles (from lowest score of 1 to highest score of 5). Information regarding the type of conception and pregnancy outcomes (i.e. gestational age (GA), birthweight (BW), birth length, multiplicity) was collected via midwives and clinical records of the birthing hospital for Gen2 participants from the Raine Study.

#### Fertility clinic records

For all GUHS participants, information regarding the cause of infertility, previous IVF cycles, IVF cycle of interest, medication scheme, day of embryo transfer, donor use, maternal maximum peri-ovulatory oestrogen levels and pregnancy outcomes (GA, BW, birth length, multiplicity) was collected from clinical records from Concept Fertility Centre and PIVET Medical Centre.

#### Midwives notification system

Additional information regarding pregnancy outcomes for GUHS participants was collected from the Midwives’ Notification System from the Western Australian Department of Health. This was used in cases of missing pregnancy outcomes from fertility clinics as fertility clinics do not always hold information regarding pregnancy outcomes.

### Ethical approvals

The GUHS received ethical approval from the Human Research Ethics Office (HREC) of University of Western Australia (RA/4/1/5860) and from the Human Research Ethics Committee of the Department of Health in Western Australia (project number 2013/25). Informed and written consent was obtained from participating families at each follow-up. Each Raine Study follow-up was approved by the HREC of the University of Western Australia.

### Statistical analysis

Statistical analyses were performed for males and females separately, to account for sex differences in cardiometabolic risk factors. Supplementary analyses on pooled male and female data were also performed to increase statistical power. We have reported the *P*-values for the total sample for outcomes that were not statistically significant in sex-specific analyses, but in the same direction as the total sample.

Univariate comparisons between GUHS and Raine participants were evaluated by Student’s *t*-test and Mann–Whitney *U* test for continuous data, and Chi^2^ or Fisher’s exact test for categorical data. Adjusted analyses were performed using generalized estimating equations with individual families modelled as random effects and adjusted for: non-singleton, BW (z-score), GA, BMI, smoking, alcohol consumption and parental cardiovascular status. Analyses of PWV and AI were additionally adjusted for standing height (m). Waist circumference was adjusted for all covariates but BMI, and additionally adjusted for standing height (m). All female analyses were also adjusted for use of oral contraceptives during the past 6 months. Univariate and adjusted subgroup analyses in male and female singletons were also performed for outcomes that indicated significant differences in the overall sample. Box-Cox transformations were performed when normality assumption was not met, and the estimated back-transformed means and their 95% CI were reported. All statistical analyses were performed using SPSS version 25.0. Armonk, NY: IBM Corp. *P* < 0.05 was considered significant. No adjustment for multiple testing was applied in accordance with the recommendations of the American Statistical Association (Lew, 2016; Wasserstein and Lazar, 2016).

## Results

Cardiometabolic profiles were compared between a total of 163 adolescents conceived with ART from the GUHS and 1457 controls conceived without ART from the Raine Study Gen2, who completed at least one component of cardiometabolic assessments. Adiposity measures, BP and heart rate assessments were completed by N = 162 and N = 1248, biochemistry analyses by N = 151 and N = 1264, liver ultrasonography by N = 136 and N = 1164 and SphygmoCor analyses by N = 158 and N = 1196. Demographic characteristics for males and females are shown in Table I. As expected, GUHS males and females had a significantly lower BW and GA, a higher percentage of preterm birth (PTB) and higher plurality. Both cohorts only included one set of triplets. GUHS males and females were significantly younger at assessment, had a lower BMI, and were less likely to consume alcohol or to smoke. A greater percentage of GUHS females had been using oral contraceptives in the preceding 6 months. Parent cardiovascular status appeared less favourable in the ART cohort, though it did not reach statistical significance. Maternal ethnicity did not differ significantly between the cohorts. The cohorts were from a similar socio-economic background and exercise frequency was similar.

**Table I:** Socio-demographic and anthropometric characteristics for males and females separately.

Demographics	ART males	Non-ART males	*P*-value	ART females	Non-Art females	*P*-value
**N**	**81**	**735**		**82**	**722**	
**Birth characteristics**						
**Birthweight** (g)	3260 (2758–3583)	3420 (3085–3733)	**0.004**	3125 (2800–3541)	3300 (2964–3630)	
	[980–4445]	[915–5550]		[970–4620]	[750–4770]	**0.030**
**Birthweight** (z-score)	0.0 (–0.7 to 0.6)	0.0 (−0.6 to 0.5)	0.937	0.1 (−0.4 to 0.8)	0.1 (−0.5 to 0.7)	
	[−2.4 to 5.3)	[−2.6 to 3.7]		[−2.3 to 8.4]	[−2.6 to 3.4]	0.427
**Birth length** (cm)	49.0 (47.0–51.0)	49.5 (48.0–51.0)	**0.014**	49.0 (47.0–51.0)	49.0 (47.0–50.0)	
	[35.0–55.0]	[33.0–56.0]		[34.0–55.0]	[30.5–57.0]	0.889
**Gestational age** (weeks)	38.3 (36.7–40.0)	39.6 (38.4–40.6)	**<0.001**	38.7 (37.0–39.7)	39.6 (38.4–40.6)	
	[26.9–42.0]	[28.7–42.9]		[27.9–42.3]	[23.6–42.7]	**<0.001**
**PTB**						
Yes	**22 (27.2%)**	**60 (8.2%)**	**<0.001**	**19 (23.2%)**	**81 (11.2%)**	**<0.001**
Missing	5 (6.2%)	1 (0.1%)		6 (7.3%)	2 (0.3%)	
**Non**–**singleton**						
Yes	**17 (21%)**	**23 (3.2%)**	**<0.001**	**16 (19.5%)**	**32 (4.4%)**	**<0.001**
Missing	0 (0.0%)	0 (0.0%)		0 (0.0%)	0 (0.0%)	
**Adolescent characteristics**						
**Age**	16.8 (16.5–17.5)	17.0 (16.9–17.1)		16.9 (16.4–17.5)	17.0 (16.9–17.2)	
	[15.6–19.0]	[16.0–18.7]	**0.003**	16.1–19.3	14.2–18.9	**0.018**
**Height** (m)	1.78 (1.73–1.82)	1.78 (1.74–1.83)		1.70 (1.63–1.75)	1.66 (1.62–1.70)	
	[1.61–1.99]	[1.57–1.99]	0.460	[1.51–1.85]	[1.36–1.87]	**<0.001**
**Weight** (kg)	66.6 (61.3–73.3)	70.0 (62.6–78.6)		61.0 (54.8–67.8)	61.5 (55.4–68.2)	
	[50.0–104.5]	[43.6–134.4]	**0.022**	[45.2–104.4]	[41.1–137.9]	0.687
**BMI** (kg/m^2^)	20.8 (19.5–23.4)	21.9 (19.9–24.1)		21.4 (19.7–23.1)	22.2 (20.1–24.5)	
	[16.0–32.8]	[14.3–42.4]	**0.036**	[16.5–34.6]	[15.0–50.2]	**0.032**
**BMI—categorical** (kg/m^2^)						
Underweight ≤16.99	2 (2.5%)	13 (1.8%)		1 (1.2%)	14 (1.9%)	
Normal 17.00–24.99	68 (84.0%)	492 (66.9%)	0.372	**70 (85.4%)**	**465 (64.4%)**	0.125
Overweight 25.00–29.99	9 (11.1%)	78 (10.6%)		8 (9.8%)	88 (12.2%)	
Obese ≥ 30.00	2 (2.5%)	46 (6.3%)		2 (2.4%)	52 (7.2%)	
Missing	0 (0.0%)	106 (14.4%)		1 (1.2%)	103 (14.3%)	
**Exercise outside of school/work**						
0–1/2 h/week	19 (23.5%)	102 (13.9%)		17 (20.7%)	157 (21.7%)	
1–3 h/week	31 (38.3%)	278 (37.8%)	0.225	37 (45.1%)	327 (45.3%)	
≥ 4 h/week	31 (38.3%)	261 (35.5%)		21 (25.6%)	158 (21.9%)	0.806
Missing	0 (0.0%)	94 (12.8%)		7 (8.5%)	80 (11.1%)	
**Alcohol—**consumption in past 6 months						
Yes	**25 (30.9%)**	**390 (53.1%)**		**14 (17.1%)**	**415 (57.5%)**	
Missing	**1 (1.2%)**	**129 (17.6%)**	**<0.001**	**8 (9.8%)**	**110 (15.2%)**	**<0.001**
**Current smoking**						
Yes	**5 (6.2%)**	**83 (11.3%)**		**2 (2.4%)**	**109 (15.1%)**	
Unknown	**0 (0.0%)**	**124 (16.9%)**	**<0.001**	**3 (3.7%)**	**103 (14.3%)**	**<0.001**
**Oral contraceptive use in past 6 months**						
Yes				**18 (22.0%)**	**15 (2.1%)**	**<0.001**
Missing	N/A	N/A		**6 (7.3%)**	**54 (7.5%)**	
**Maternal ethnicity**						
Caucasian	79 (97.5%)	651 (88.6%)		77 (93.9%)	636 (88.1%)	
Unknown	0 (0.0%)	13 (1.8%)	0.165	0 (0.0%)	14 (1.9%)	0.403
**Parent cardiovascular risk**						
Yes	53 (65.4%)	359 (48.8%)		47 (57.3%)	317 (43.9%)	
Missing	5 (6.2%)	169 (23.0%)	0.281	13 (15.9%)	205 (28.4%)	0.274
**Family SES based on SEIFA**						
1st Quintile	0 (0.0%)	16 (2.3%)		3 (3.7%)	19 (2.6%)	
2nd Quintile	12 (14.8%)	42 (5.7%)	0.116	13 (15.9%)	40 (5.5%)	0.161
3rd Quintile	15 (18.5%)	86 (11.7%)		12 (14.6%)	91 (12.6%)	
4th Quintile	12 (14.8%)	101 (13.7%)		11 (13.4%)	93 (12.9%)	
5th Quintile	42 (51.9%)	311 (42.3%)		40 (48.8%)	289 (40.0%)	
Missing	0 (0.0%)	179 (24.4%)		3 (3.7%)	190 (26.3%)	

Data are shown as median (25–75th percentile) [range] and as N (%). Significant outcomes (*P*<0.05) are presented in bold.

Number of participants includes participants who completed at least one cardiometabolic assessment.

PTB, preterm birth (<37 weeks gestational age); SES, socio-economic status; SEIFA, Socio-Economic Indexes for Areas: Index of Advantage-Disadvantage (lowest score of 1 to the highest score of 5); N/A, not applicable.

### Adiposity

Results of adiposity measures for males and females are shown in Table II. In adjusted analyses, BMI was lower in GUHS adolescents in both the total sample (*P* = 0.005), as well as in females (22.1 (20.9–23.3) vs 23.3 (22.4–25.5), *P* = 0.014). In males, BMI did not significantly differ between the cohorts (22.0 (21.0–23.1) vs 22.7 (21.8–23.8) kg/m^2^, *P* = 0.097). Overall, fewer GUHS than Gen2 adolescents were obese (BMI ≥30 kg/m^2^) (2.5% vs 7.9%, *P* = 0.031). This trend was also seen in males and female alone, albeit was non-significant due to lacking power (males: 2.5% vs 7.3%, *P* = 0.372; females: 2.5% vs 8.4%, *P* = 0.125). Skinfolds were significantly lower in GUHS in males and females combined (subscapular *P* = 0.002, mid-abdominal *P* = 0.009, suprailiac *P* = 0.005), but not for the triceps skinfold (*P* = 0.122). In females, the triceps (16.9 (15.7–18.3) vs 18.7 (17.3–20.2) mm, *P* = 0.021), subscapular (13.4 (12.4–14.7) vs 15.0 (13.8–16.5), *P* = 0.027) and mid-abdominal skinfold (19.7 (17.8–21.7) vs.23.2 (20.9–25.6), *P* < 0.001) were lower in GUHS adolescents. The suprailiac skinfold was similar in both cohorts (15.1 (13.7–16.7) vs 16.1 (14.4–18.1) mm, *P* = 0.248). In males, there were no differences in these skinfolds between GUHS and Gen2 adolescents (8.7 (7.4–10.2) vs 9.2 (8.1–10.5) mm, *P* = 0.326; 9.5 (8.0–11.4) vs 9.7 (8.2–11.6) mm, *P* = 0.699; 10.9 (8.4–13.9) vs 12.2 (10.2–14.6) mm, *P* = 0.171; and 8.4 (6.7–10.6) vs 9.4 (8.0–11.1) mm, *P* = 0.178, respectively). Waist circumference was significantly lower in GUHS males and females compared with the Gen2 adolescents (78.1 (75.5–80.8) vs 81.3 (79.1–83.7) cm, *P* = 0.008 and 76.7 (72.8–81.1) vs 83.3 (78.8–88.5) cm, *P* = 0.007, respectively).

**Table II:** Adiposity measures compared between adolescents conceived with ART (GUHS) and without ART (Gen2), depicted separately for males and females.

Males	ART	Non-ART	Univariate *P*-value	ART	Non-ART	Adjusted *P*-value*
**BMI** (kg/m^2^)	20.8	21.9				
	(19.5–23.4)	(19.9–24.1)		22.0	22.7	
	[16.0–32.8]	[14.3–42.4]	**0.036**	(21.0–23.1)	(21.8–23.8)	0.097
**Waist circumference** (cm)	75.4	77.8				
	(71.6–80.3)	(73.7–84.6)		78.1	81.3	
	[55.9–108.7]	[55.0–142.3]	**0.001**	(75.5–80.8)	(79.1–83.7)	**0.008**
**Skinfolds**						
Triceps (mm)	8.2	9.2				
	(6.5–11.0)	(7.2–12.73)		8.7	9.2	
	[3.1–27.0]	[4.0–41.0]	**0.014**	(7.4–10.2)	(8.1–10.5)	0.326
Subscapular (mm)	9.3	10.0				
	(7.9–11.1)	(8.1–13.8)		9.5	9.7	
	[4.1–34.2]	[4.9–41.0]	**0.037**	(8.0–11.4)	(8.2–11.6)	0.699
Mid-abdominal (mm)	12.3	14.0				
	(8.3–18.5)	(9.2–22.6)		10.9	12.2	
	[5.2–39.2]	[4.7–41.0]	0.066	(8.4–13.9)	(10.2–14.6)	0.171
Suprailiac (mm)	8.1	9.8				
	(6.1–11.1)	(6.8–16.3)		8.4	9.4	
	[4.0–38.6]	[3.4–41.0]	**0.015**	(6.7–10.6)	(8.0–11.1)	0.178

**Females**	**ART**	**Non-ART**	**Univariate *P*-value**	**ART**	**Non-ART**	**Adjusted *P*-value** **
**BMI—continuous** (kg/m^2^)	21.4	22.2				
	(19.7–23.1)	(20.1–24.5)		22.1	23.3	
	[16.5–34.6]	[15.0–50.2]	**0.032**	(20.9–23.3)	(22.4–25.5)	**0.014**
**Waist circumference** (cm)	72.9	75.5				
	(68.4–79.1)	(69.9–82.5)		76.7	83.3	
	[61.1–103.7]	[38.0–138.7]	**0.019**	(72.8–81.1)	(78.8–88.5)	**0.007**
**Skinfolds**						
Triceps (mm)	16.3	18.4				
	(13.7–20.5)	(14.9–22.6)		16.9	18.7	
	[7.1–31.0]	[6.7–41.0]	**0.017**	(15.7–18.3)	(17.3–20.2)	**0.021**
Subscapular (mm)	12.1	14.3				
	(9.7–15.6)	(11.2–19.2)		13.4	15.0	
	[7.1–31.0]	[5.1–41.0]	**<0.001**	(12.4–14.7)	(13.8–16.5)	**0.027**
Mid-abdominal (mm)	18.4	23.9				
	(14.5–24.4)	(18.4–30.3)		19.7	23.2	
	[7.5–33.6]	[5.4–41.0]	**<0.001**	(17.8–21.7)	(20.9–25.6)	**<0.001**
Suprailiac (mm)	14.1	17.4				
	(10.1–18.7)	(12.5–22.9)		15.1	16.1	
	[6.2–34.6]	[2.2–41.0]	**<0.001**	(13.7–16.7)	(14.4–18.1)	0.248

Continuous outcomes are presented as medians (25th–75th percentile) [range] for univariate analyses and as mean (95% CI) after covariate adjustment. Significant outcomes (*P*<0.05) are presented in bold.

* Adjusted for: non-singleton, gestational age, birthweight z-score, BMI, smoking, alcohol intoxication and parental cardiovascular status.

** Adjusted for: non-singleton, gestational age, birthweight z-score, BMI, smoking, alcohol intoxication, oral contraceptive use and parental cardiovascular status. Waist circumference was adjusted for height instead of BMI.

GUHS, Growing Up Healthy Study; Gen2, Generation 2.

### Fasting serum biochemistry

Mean fasting serum biochemistry levels for males and females are shown in Table III. All mean serum fasting parameters fell within the normal range in both cohorts in males and females, with no significant differences in glucose, insulin, HOMA-IR, LDL-C, TC, non-HDL-C, ALT and hs-CRP in both sexes. In females, triglycerides were lower in GUHS adolescents (1.0 (0.8–1.1) vs 1.2 (1.0–1.3) mmol/l. *P* = 0.029), but there were no differences in the HDL-C and TC/HDL-C ratio. In males, there was no significant difference in triglycerides, but HDL-C was increased in GUHS males (1.1 (1.0–1.2) vs 1.0 (1.0–1.1) mmol/l, *P* = 0.004) and the TC/HDL-C ratio was lower (3.2 (2.6–3.9) vs 3.6 (3.0–4.4), *P* = 0.036). The same trend for the TC/HDL-C ratio was evident in the total sample (*P* = 0.006), but not in females alone (3.1 (2.9–3.4) vs 3.4 (3.0–3.7), *P* = 0.219). Contrary to our hypothesis, the Gen2 cohort had a higher BMI, therefore lipid analyses were also conducted without BMI adjustment. In these analyses, the outcomes remained unchanged (data not shown).

**Table III:** Fasting serum biochemistry markers compared between adolescents conceived with ART (GUHS) and without ART (Gen2), depicted separately for males and females.

Males	ART	Non-ART	Univariate *P*-value	ART	Non-ART	Adjusted *P*-value*
**Glucose** (mmol/l)	4.8	4.8				
	(4.5–5.1)	(4.6–5.1)		4.9	4.8	
	[4.2–6.0]	[3.2–14.4]	0.978	(4.7–5.0)	(4.7–4.9)	0.716
**Insulin** (mU/l)	6.0	7.1				
	(5.0–9.0)	(4.6–10.6)		7.2	7.2	
	[3.0–13.0]	[1.9–88.9]	0.336	(6.1–8.4)	(6.2–8.4)	0.881
**HOMA-IR**	1.4	1.5				
	(1.2–2.0)	(1.0–2.3)		1.6	1.6	
	[0.6–3.0]	[0.3–24.5]	0.437	(1.3–1.8)	(1.3–1.8)	0.947
**Triglycerides** (mmol/l)	0.9	0.9				
	(0.7–1.1)	(0.7–1.3)		1.0	1.0	
	[0.5–2.7]	[0.4–6.0]	0.286	(0.9–1.2)	(0.9–1.2)	0.394
**LDL-C** (mmol/l)	2.3	2.2				
	(1.8–2.6)	(1.8–2.6)		2.0	2.1	
	[1.3–4.2]	[0.1–4.8]	0.660	(1.4–2.6)	(1.5–2.7)	0.446
**HDL-C** (mmol/l)	1.3	1.2				
	(1.1–1.4)	(1.0–1.4)		1.1	1.0	
	[1.0–2.0]	[0.5–2.3]	**0.006**	(1.0–1.2)	(1.0–1.1)	**0.004**
**TC** (mmol/l)	3.9	3.9				
	(3.5–4.4)	(3.5–4.4)		3.6	3.8	
	[2.9–5.8]	[1.6–7.2]	0.548	(3.0–4.3)	(3.2–4.4)	0.467
**Non-HDL-C** (mmol/l)	2.7	2.6				
	(2.3–3.0)	(2.2–3.2)		2.5	2.7	
	[1.6–4.7]	[0.6–6.0]	0.974	(1.8–3.2)	(2.1–3.4)	0.228
**TC/HDL ratio**	3.2	3.3				
	(2.7–3.5)	(2.8–3.8)		3.2	3.6	
	[2.0–5.3]	[1.6–7.5]	0.091	(2.6–3.9)	(3.0–4.4)	**0.036**
**Hs-CRP** (mg/l)***	0.4	0.4				
	(0.2–1.1)	(0.2–1.1)		0.5	0.4	
	[0.1–5.3]	[0.1–9.7]	0.972	(0.4–0.7)	(0.3–0.5)	0.24
**ALT** (U/l)	22.0	21.0				
	(16.0–30.0)	(16.0–29.0)		16.4	16.1	
	[11.0–66.0]	[4.0–215.0]	0.237	(11.6–23.5)	(12.1–21.7)	0.828

**Females**	**ART**	**Non-ART**	**Univariate *P*-value**	**ART**	**Non-ART**	**Adjusted *P*-value** **
**Glucose** (mmol/l)	4.7	4.6				
	(4.4–4.9)	(4.4–4.9)		4.6	4.6	
	[3.9–5.6]	[3.8–12.6]	0.580	(4.5–4.8)	(4.4–4.7)	0.508
**Insulin** (mU/l)	7.5	7.7				
	(6.0–9.0)	(5.2–11.0)		7.2	7.3	
	[3.0–20.0]	[1.9–238.0]	0.661	(6.2–8.3)	(6.0–9.0)	0.802
**HOMA-IR**	1.5	1.6				
	(1.2–1.9)	(1.1–2.3)		1.5	1.5	
	[0.6–4.7]	[0.4–60.3]	0.847	(1.3–1.7)	(1.2–1.8)	0.956
**Triglycerides** (mmol/l)	0.9	0.9				
	(0.7–1.1)	(0.7–1.2)		1.0	1.2	
	[0.4–2.2]	[0.3–5.5]	0.111	(0.8–1.1)	(1.0–1.3)	**0.029**
**LDL-C** (mmol/l)	2.4	2.4				
	(2.0–2.8)	(2.0–2.8)		2.5	2.7	
	[1.1–4.0]	[0.3–5.2]	0.950	(2.2–2.8)	(2.4–3.0)	0.252
**HDL-C** (mmol/l)	1.5	1.4				
	(1.3–1.6)	(1.2–1.6)		1.4	1.4	
	[0.9–2.0]	[0.6–2.8]	**0.01**	(1.3–1.5)	(1.3–1.5)	0.69
**TC** (mmol/l)	4.3	4.2				
	(3.8–4.8)	(3.8–4.7)		4.4	4.6	
	[2.8–6.2]	[2.2–7.3]	0.693	(4.1–4.7)	(4.4–4.9)	0.156
**Non-HDL-C** (mmol/l)	2.8	2.8				
	(2.4–3.3)	(2.4–3.3)		3.0	3.2	
	[1.5–4.7]	[0.4–5.8]	0.462	(2.7–3.3)	(3.0–3.5)	0.16
**TC/HDL-C ratio**	3.0	3.1				
	(2.5–3.4)	(2.6–3.7)		3.1	3.4	
	[1.8–4.5]	[1.2–6.6]	0.053	(2.9–3.4)	(3.0–3.7)	0.219
**Hs-CRP** (mg/l)***	0.6	0.8				
	(0.2–1.1)	(0.3–2.0)		1.1	1.3	
	[0.1–8.4]	[0.1–9.6]	**0.010**	(0.8–1.6)	(0.9–1.9)	0.434
**ALT** (U/l)	17.0	16.0				
	(13.5–24.0)	(12.0–22.0)		17.2	15.9	
	[7.0–35.0]	[2.0–209.0]	0.097	(14.9–20.0)	(13.5–18.7)	0.267

Continuous outcomes are presented as medians (25th–75th percentile) [range] for univariate analyses and as mean (95% CI) after covariate adjustment. Significant outcomes (*P*<0.05) are presented in bold.

* Adjusted for: non-singleton, gestational age, birthweight (z-score), BMI, smoking, alcohol intoxication and parental cardiovascular status.

** Adjusted for: non-singleton, gestational age, birthweight (z-score), BMI, smoking, alcohol consumption, oral contraceptive use, and parental cardiovascular status.

*** Excluded hs-CRP >10 mg/l.

HOMA-IR, homeostatic model assessment for insulin resistance; TC, total cholesterol; LDL-C, low-density lipoprotein cholesterol; HDL-C, high-density lipoprotein cholesterol; hs-CRP, high-sensitivity C-reactive protein; GUHS, Growing Up Healthy Study; ALT, alanine aminotransferase; Gen2, Generation 2.

### Abdominal ultrasonography

Results from liver ultrasonography are shown in Table IV. One female GUHS participant, who classified as having NAFLD based on ultrasound characteristics, was excluded from NAFLD analysis, due to long-term medication use potentially affecting the liver. There was no significant difference in the prevalence of NAFLD in males and females combined or separately, although NAFLD appeared less prevalent in GUHS adolescents, particularly in females (females: 12.5% vs 19.7%, odds ratio (OR) 0.64 (0.30–1.37), *P* = 0.180, males: 9.4% vs 10.9%, OR 0.50 (0.70–1.71), *P* = 0.413). No differences in prevalence of hepatic steatosis were demonstrated between the cohorts (males: OR 0.55 (0.61–1.71), *P* = 0.354, females: OR 0.50 (0.38–1.37), *P* = 0.268). GUHS adolescents had significantly less subcutaneous adipose tissue in the total cohort (*P* < 0.001), which was also evident in females (9.4 (8.4–10.5) vs 17.9 (15.7–20.3) mm, *P* < 0.001). The same effect, although not statistically significant, was seen in males (11.7 (7.8–17.4) vs 15.3 (11.0–21.4) mm, *P* = 0.130). GUHS adolescents had a significant increase in visceral adipose tissue in the total cohort (*P* < 0.001), which was also evident in males (44.7 (40.8–48.8) vs 36.3 (33.9–38.9) mm, *P* < 0.001). An analogous effect, approaching statistical significance, was seen in females (34.4 (29.9–39.0) vs 29.7 (25.2–34.4) mm, *P* = 0.088). No difference in pre-peritoneal adipose tissue thickness was demonstrated.

**Table IV:** Abdominal ultrasound measures compared between adolescents conceived with ART (GUHS) and without ART (Gen2), depicted separately for males and females.

Males		ART	Non-ART	Univariate *P*-value	ART	Non-ART	Adjusted *P*-value*
**NAFLD**, N (%)		6 (9.4%)	64 (10.9%)	0.714	OR 0.50 (0.70–1.71)		0.413
**Steatosis severity score**	No hepatic steatosis	58 (90.6%)	528 (89.6%)				
	Mild hepatic steatosis	4 (6.3%)	29 (4.9%)	0.672	OR 0.55*** (0.61–1.71)		0.354
	Moderate hepatic steatosis	2 (3.1%)	32 (5.4%)				
**Abdominal adipose tissue thickness**							
Subcutaneous (mm)		7.0	11.0				
		(5.0–9.3)	(7.4–17.3)		11.7	15.3	
		[3.0–58.0]	[2.0–58.0]	**<0.001**	(7.8–17.4)	(11.0–21.4)	0.13
Visceral (mm)		44.0	35.0				
		(35.8–49.3)	(28.0–42.0)		44.7	36.3	
		[16.0–87.0]	[6.0–84.0]	**<0.001**	(40.8–48.8)	(33.9–38.9)	**<0.001**
Pre-peritoneal Adipose (mm)		6.0	6.0				
		(4.0–9.3)	(4.0–9.0)		5.8	5.2	
		[2.0–60.0]	[0.0–43.0]	0.492	(4.6–7.3)	(4.4–6.2)	0.279

Females		ART	Non-ART	Univariate *P*-value	ART	Non-ART	Adjusted *P*-value**
**NAFLD**, N (%)		9 (12.5%)	113 (19.7%)	0.144	OR 0.64 (0.30–1.58)		0.180
**Steatosis severity score**	No hepatic steatosis	62 (86.1%)	463 (80.5%)				
	Mild hepatic steatosis	8 (11.1%)	73 (12.7%)	0.369	OR 0.50*** (0.38–1.37)		0.268
	Moderate hepatic steatosis	2 (2.8%)	39 (6.8%)				
**Abdominal adipose tissue thickness**							
Subcutaneous adipose tissue (mm)		10.0	18.0				
		(7.0–13.0)	(13.0–26.0)		9.4	17.9	
		[3.0–24.0]	[0.00–74.0]	**<0.001**	(8.4–10.5)	(15.7–20.3)	**<0.001**
Visceral Adipose tissue (mm)		37.0	29.5				
		(29.0–43.0)	(24.0–35.8)		34.4	29.7	
		[9.0–59.0]	[2.5–66.0]	**<0.001**	(29.9–39.0)	(25.2–34.4)	0.088
Pre-peritoneal adipose tissue (mm)		9.0	8.0				
		(6.3–12.8)	(7.0–11.0)		14.5	15.1	
		[3.0–48.0]	[2.0–43.0]	0.295	(11.2–18.8)	(11.8–19.2)	0.810

Continuous outcomes are presented as medians (25th–75th percentile) [Range] for univariate analyses and as mean (95% CI) after covariate adjustment. Categorical univariate outcomes presented as percentages and categorical adjusted outcomes as OR (95% CI) relative to Gen2. Significant outcomes (*P*<0.05) are presented in bold.

* Adjusted for: non-singleton, gestational age, birthweight (z-score), BMI, smoking, alcohol intoxication and parental cardiovascular status.

** Adjusted for: non-singleton, gestational age, birthweight (z-score), BMI, smoking, alcohol intoxication, oral contraceptive use and parental cardiovascular status.

*** OR refers to any hepatic steatosis vs no hepatic steatosis.

NAFLD, non-alcoholic fatty liver disease; GUHS, Growing Up Healthy Study; Gen2, Generation 2; OR, odds ratio.

### Arterial stiffness

SphygmoCor results for males and females are presented in Table V. PWV was significantly lower in the GUHS males and females combined (*P* < 0.001) and in males alone (males: 5.8 (5.5–6.2) vs 6.3 (6.0–6.6) m/s, *P* < 0.001; females: 6.2 (6.0–6.4) vs 6.3 (6.1–6.5) m/s, *P* = 0.152). AI was lower in GUHS adolescents overall and in females (−8.4 (−13.9 to −2.9) vs −2.7 (−6.2 to 0.6) %, *P* = 0.048), but not in males (−9.6 (−13.3 to −5.9) vs −9.5 (−12.3 to −6.7) %, *P* = 0.952). No differences in systolic BP, diastolic BP and heart rate were detected in the total cohort, or in males and females separately. When removing the BMI adjustment for PWV and BP analyses, the results remained the same (data not shown).

**Table V:** Arterial stiffness measures and blood pressure compared between adolescents conceived with ART (GUHS) and without ART (Gen2), depicted separately for males and females.

Males	ART	Non-ART	Univariate *P*-value	ART	Non-ART	Adjusted *P*-value*
**Mean PWV** (m/s)	6.2	6.6				
	(5.5–6.8)	(6.2–7.1)		5.8	6.3	
	[3.6–11.2]	[4.7–9.4]	**<0.001**	(5.5–6.2)	(6.0–6.6)	**<0.001**
**Mean heart rate corrected AI** (%)						
	−10.5	−9.5				
	(−20.1 to −2.0)	(−17.5 to −2.5)		−9.6	−9.5	
	[−47.0 to 22.0]	[−44.0 to 29.5]	0.317	(−13.3 to −5.9)	(−12.3 to −6.7)	0.952
**Mean systolic pressure** (mmHg)						
	118	119				
	(112–125)	(113–126)		120	120	
	[94–144]	[83–153]	0.589	(117–123)	(117–123)	0.897
**Mean diastolic pressure** (mmHg)						
	58	59				
	(56–63)	(55–63)		56	57	
	[45–88]	[43–82]	0.735	(50–63)	(51–63)	0.798
**Mean heart rate** (BPM)	60	62				
	(55–67)	(56–69)		62	64	
	[38–95]	[38–126]	0.167	(59–65)	(61–66)	0.258

**Females**	**ART**	**Non-ART**	**Univariate *P*-value**	**ART**	**Non-ART**	**Adjusted *P*-value** **
**Mean PWV** (m/s)	6.0	6.3				
	(5.5–6.6)	(5.9–6.7)		6.2	6.3	0.152
	[4.9–7.7]	[4.4–8.6]	**<0.001**	(6.0–6.4)	(6.1–6.5)	
**Mean heart rate corrected AI** (%)						
	−10.5	−6.5				
	(−17.3 to −2.8)	(−13.5 to 1.0)		−8.4	−2.7	
	[−51.0 to 19.0]	[−29.5 to 26.5]	**0.006**	(−13.9 to −2.9)	(−6.2 to 0.6)	**0.048**
**Mean systolic pressure** (mmHg)						
	113	110				
	(107–118)	(103–116)		113	110	
	[89–133]	[81–148]	0.245	(110–115)	(107–113)	0.060
**Mean diastolic pressure** (mmHg)						
	59	59				
	(55–63)	(55–64)		57	58	
	[41–77]	[44–84]	0.517	(54–60)	(55–62)	0.649
**Mean heart rate** (BPM)	67	67				
	(60–73)	(61–74)		56	60	
	[45–91]	[37–115]	0.255	(50–63)	(55–65)	0.295

Continuous outcomes are presented as medians (25th–75th percentile) [range] for univariate analyses and as mean (95% CI) after covariate adjustment. Significant outcomes (*P*<0.05) are presented in bold.

* Adjusted for: non-singleton, gestational age, birthweight (z-score), BMI, smoking, alcohol intoxication and parental cardiovascular status.

** Adjusted for: non-singleton, gestational age, birthweight (z-score), BMI, smoking, alcohol intoxication, oral contraceptive use and parental cardiovascular status. PWV and AI analyses additionally adjusted for height.

PWV, pulse wave velocity; AI, augmentation index; GUHS, Growing Up Healthy Study; Gen2, Generation 2.

### Sensitivity analysis

For outcomes that were significant in males or females in the total cohort, analyses were additionally run for singleton males and females. The direction of these results remained the same, and the magnitude of the effects was even more pronounced in singletons. Waist circumference in males lost significance (data not shown).

## Discussion

In the present study of 17-year-old adolescents, we report mostly similar or more favourable cardiometabolic parameters in ART compared to non-ART conceived adolescents. Whilst the data show statistically significant differences, all mean parameters fall within normal clinical ranges.

This study has shown that adolescents conceived after ART had a lower BMI, thinner waist circumference and thinner skinfold thickness, lower arterial stiffness and a slightly more favourable blood lipid profile in males (higher HDL-C and lower TC/HDL-C ratio), as well as lower triglycerides in females. NAFLD tended to be more prevalent in the non-ART cohort, particularly in females (males: 9.4% vs 10.9%, females: 12.5% vs 19.7%), however, this did not reach statistical significance, likely due to lack of statistical power. These findings are reassuring for ART conceived offspring and are potentially in line with emerging epigenetic studies demonstrating that reported epigenetic alterations in ART newborns, potentially leading to cardiometabolic differences (Fleming *et al.*, 2018), are mitigated by adolescence (Novakovic *et al.*, 2019; Penova-Veselinovic *et al.*, 2021). A recent study compared the epigenome profiles of the adolescents of the present study (GUHS) to those of the same comparator cohort (the Raine Study Gen2), using the ‘epigenome-wide DNA methylation association studies’ approach, and demonstrated no significant differences in the DNA methylation profiles between the cohorts (Penova-Veselinovic *et al.*, 2021). The current findings suggest that the previously reported differences in cardiometabolic health in children conceived with and without ART might be resolved by adolescence.

It has been suggested that the changes and improvements in ART practices over the past decades, with milder stimulation protocols, single embryo transfer (SET) policies and improved laboratory techniques, could potentially translate to fewer epigenetic alterations, and therefore less cardiometabolic differences between offspring conceived with and without ART (Berntsen *et al.*, 2019). This would explain why a large meta-analysis reported an increase in systolic and diastolic BP in older cohorts (1990–1999) and not in younger cohorts (>2000; Guo *et al.*, 2017). Furthermore, a reduction in multiple pregnancies, following the introduction of SET policies in many countries, has reduced the number of low BW (LBW) and PTB in pregnancies following ART. LBW and PTB are known risk factors for future metabolic syndrome (Johansson *et al.*, 2005; Kaijser *et al.*, 2008; de Jong *et al.*, 2012). Therefore, a reduction in these perinatal risk factors could also translate to a decrease in reported cardiometabolic differences. Despite the higher rates of PTB and relatively lower BW in the present ART cohort, there was no increase in most cardiometabolic risk factors assessed. Because of the high percentage of multiples in the ART cohort of the present study (males: 21% vs 3.2%, *P* < 0.001; females: 19.5% vs 4.4%, *P* < 0.001), we ran further analyses on male and female singletons separately, which did not alter the findings.

Several recent studies have reported similar results to those found in our study. Halliday *et al.* (2019) investigated adults aged 22–35 years and reported no evidence of an increased vascular or cardiometabolic risk in adults conceived after ART. In a large Scandinavian registry study, no difference in the risk of cardiovascular disease and type 2 diabetes was observed between offspring conceived after ART and controls. A small but statistically significant increase in obesity was reported in the ART offspring (Norrman *et al.*, 2021). Another study reported no difference in markers of metabolic syndrome, except for a lower HDL-C in men aged 18–22 conceived after ICSI (Belva *et al.*, 2018). Whilst reporting an increase in systolic and diastolic BP, the meta-analysis by Guo *et al.* (2017) also reported comparable fasting insulin levels, low-density lipoproteins and BMI between offspring conceived with and without ART. A small study from New Zealand demonstrated a slightly more favourable lipid profile (higher HDL-C and lower LDL-C and triglycerides) in children conceived after IVF (Miles *et al.*, 2007).

The present study did demonstrate an increase in visceral adipose tissue in adolescents conceived after ART (significant in the total cohort and in males). Visceral adipose tissue is the most unfavourable type of adipose tissue and is correlated with the metabolic syndrome, which in turn is a predictor of cardiovascular health (Wajchenberg, 2000). Several studies have reported an increase in body fat in children conceived after ART (Ceelen *et al.*, 2007; Belva *et al.*, 2012). Even though the ART cohort in our study have less overall adiposity and obesity, this did not translate to beneficial metabolic effects on triglycerides in males, insulin resistance and hs-CRP, which may be related to the greater visceral fat mass. Rapid infant catch-up growth has been associated with an increase in abdominal adiposity and visceral fat mass (Demerath *et al.*, 2009). Premature children and children who are born small for GA are particularly prone to rapid catch-up growth with negative health effects in adult life (Kerkhof and Hokken-Koelega, 2012). As expected, in our study, PTB and LBW were significantly more prevalent in the ART cohort, although adjusting for these and other covariates did not alter the reported increase in visceral fat tissue in the ART cohort, apart from losing significance in females. Interestingly, the increase in visceral fat tissue was also seen in the sub-analyses of singleton males and females. The fact that the present study reports no unfavourable cardiometabolic outcomes apart from an increase in visceral adiposity (significant in the total cohort and males), could mean that cardiometabolic differences are potentially masked during adolescence by various factors, such as a generally unhealthier lifestyle among some adolescents, and differences could perhaps become more apparent again in adulthood. It would, therefore, be of great interest to assess these, and other adolescents, once they reach adulthood, to investigate if indeed the reported differences in childhood are reduced or have even disappeared by adulthood.

Future studies could further investigate whether there are potential differences in metabolic risk in subsets of offspring according to the different causes of parental infertility and/or parental cardiometabolic health. It has been suggested for example that unfavourable cardiometabolic profiles of parents could explain the unfavourable cardiometabolic health in ART children (Mulder *et al.*, 2020). It would be valuable for future studies to investigate the cardiometabolic health of ICSI offspring separately. ICSI as opposed to IVF, is more invasive, with elimination of natural selection and mechanical manipulation of sperm and oocytes (Berntsen *et al.*, 2019), and concern has been raised about passing on more poor-quality sperm (Davies *et al.*, 2012). All these factors could lead to an increase of epigenetic alterations following ICSI and therefore the cardiometabolic health of ICSI offspring is of particular interest. Potentially, cardiometabolic differences reported by other studies could be partly explained by a large proportion of ICSI participants. At the time of the conceptions in this study, ICSI was used infrequently. Furthermore, it would be of interest to compare FET with fresh transfers (ET). FET has been positively associated with a reduction in LBW and PTB, which are known risk factors for metabolic syndrome (Zhao *et al.*, 2016; Maheshwari *et al.*, 2018). As adverse pregnancy outcomes such as PTB and LBW, which are often seen in multiple pregnancies, have been associated with the metabolic syndrome later in life, larger studies including only ART singletons are of importance (Johansson *et al.*, 2005; Kaijser *et al.*, 2008; de Jong *et al.*, 2012).

The main strength of this study is the study design, where the adolescents conceived after ART replicated the same assessments previously completed by their well-characterized counterparts conceived without ART. In combination with the relatively static population of Western Australia, this is a unique study design that would be difficult to replicate elsewhere in Australia or internationally. Additionally, as it was attempted to approach all adolescents conceived through ART in Western Australia during a 10-year period via both operating fertility clinics, selection bias is reduced. A further strength is the inclusion of a wide range of cardiometabolic outcomes, presenting an extensive and comprehensive cardiometabolic profile at adolescence.

This study also has various limitations. Despite the substantial overall cohort size, our sample size did not allow for investigation of FET vs ET, ICSI vs IVF or analysis by cause of infertility; in particular underlying maternal polycystic ovary syndrome, with its known relationship to insulin resistance and metabolic syndrome would be of prime interest. Large registry studies could attempt to compare cardiometabolic health by parental cause of infertility, to assess a potential association between specific causes of infertility and cardiometabolic health of the offspring. Although we analysed males and females, as well as singleton males and females separately, some outcomes were compromised due to the relatively small sample size.

Despite the ability to adjust for some important confounding variables, there is still a risk of residual confounding as our numbers did not allow for adjustment of more variables. Some important factors not adjusted for included maternal BMI and smoking during pregnancy, obstetric complications and diet. As the cohorts appear to differ on lifestyle factors affecting cardiometabolic health, such as current smoking and alcohol consumption, their diet may also differ. Although adjustment for smoking and alcohol consumption did not greatly alter the results, residual confounding from lifestyle-related factors cannot be entirely ruled out. Furthermore, as some of these variables, particularly those regarding pregnancy (complications), were collected retrospectively from a state registry, missing data limited the ability to include them in the model. Data regarding paternal ethnicity were not recorded and ethnicity could therefore not be accounted for. Despite most data being collected in an identical manner in both cohorts, data regarding pregnancy (outcomes) were collected from fertility clinics and the MNS for the GUHS cohort and collected from midwives and clinical records from the respective birthing hospital for the Raine Study. This difference in data acquisition needs to be considered when interpreting the data. A further limitation is the time between assessments of adolescents conceived without ART from the Raine Study and those of adolescents conceived with ART from the GUHS. To replicate assessments at the same age, GUHS assessments took place between 4 and 11 years after the Raine Study assessments. Although protocols and methodologies were identical, external factors, as well as adolescent behaviour, may have changed which could have affected study results. Additionally, despite 99% of age-eligible ART adolescents completing at least one cardiovascular assessment at the 17-year follow-up, this percentage is much lower in the Raine Study (51%). This is greatly explained by a loss to follow-up in the Raine Study. As we do not have information on characteristics of the participants who were lost to follow-up, this could have affected the results. It is possible that particularly families of lower SES were lost to follow-up, which could explain the similar SES between the ART and non-ART cohort in this study at the time of assessment. Unfortunately, SES at time of pregnancy was not available, which could have introduced a bias which we could not account for. Lastly, the interpretation of results also needs to consider that multiple testing in this study could have led to some chance findings.

## Conclusion

This study with a particular focus on adolescence mostly shows a similar, and in some cases more favourable, cardiometabolic profile in offspring conceived with ART in comparison to offspring conceived without ART. Adolescents conceived with ART had a lower BMI, thinner waist circumference, thinner skinfold thickness and less arterial stiffness. Males had a more favourable blood lipid profile and females had lower triglycerides compared to their counterparts conceived without ART. However, males conceived with ART had greater visceral fat, which may suggest that potential cardiometabolic differences could be masked during adolescence and might become apparent again during adulthood. Further studies into adulthood are warranted.

## Data availability

The data underlying this article cannot be shared publicly for ethical reasons and privacy protection of the individuals that participated in the study. The data will be shared upon reasonable request to the corresponding author.

## Notes

Presented at ESHRE 2021, https://doi.org/10.1093/humrep/deab125.002
